# Photodynamic Therapy and Tumor Microenvironment-Targeting Strategies: A Novel Synergy at the Frontier of Cancer Treatment

**DOI:** 10.3390/ijms26178588

**Published:** 2025-09-03

**Authors:** Stefani Torna, Vasiliki Gkretsi, Andreas Stylianou

**Affiliations:** 1 Cancer Mechanobiology and Applied Biophysics Group, Basic and Translational Cancer Research Center, School of Sciences, European University Cyprus, Nicosia 2404, Cyprus; 2024euc1211@students.euc.ac.cy; 2 Biomedical Sciences Program, Department of Life Sciences, School of Sciences, European University Cyprus, Nicosia 2404, Cyprus; v.gkretsi@euc.ac.cy; 3 Cancer Metastasis and Adhesion Group, Basic and Translational Cancer Research Center (BTCRC), European University Cyprus, Nicosia 2404, Cyprus; 4 EUC Research Center, European University Cyprus, Nicosia 2404, Cyprus

**Keywords:** photodynamic therapy (PDT), tumor microenvironment (TME), combination therapy, hypoxia modulation, vascular normalization

## Abstract

Despite intensive worldwide research efforts and multiple available therapeutic schemes for cancer treatment, cancer still remains a challenge, rendering the need for the discovery of new therapeutic approaches imperative. Photodynamic therapy (PDT) is a novel, non-invasive anti-cancer treatment that relies on the generation of reactive oxygen species (ROS) that are cytotoxic to cancer cells. ROS are generated by the interaction between a photosensitizer (PS) drug, a light source (primarily a laser), and oxygen. Although PDT offers the advantage of using non-ionizing radiation and bears great therapeutic potential, it has not yet been widely adopted in clinical practice. This review summarizes the new developments in the use of PDT in combination with chemotherapy, immunotherapy, and radiotherapy, giving emphasis to the combination of PDT with a novel type of therapy that also takes into account the tumor microenvironment (TME) to enhance treatment efficacy. TME-targeting therapies include strategies like hypoxia modulation, vascular normalization, and immune cell reprogramming. Interestingly, when combined with PDT, these therapies can improve therapeutic outcomes while reducing side effects, and nanoparticle-based delivery systems have demonstrated the potential to enhance PDT selectivity and efficiency. This review highlights PDT’s enormous potential in treating various cancer types and underscores the need for continued exploration of combination therapies to maximize its clinical impact.

## 1. Introduction

### 1.1. Cancer and Tumor Microenvironment (TME)

Cancer is characterized by abnormal cell proliferation, eventually leading to the formation of a tumor. When the tumor becomes malignant, cells acquire the capacity to dissociate from the original tumor mass, migrate, and invade through surrounding tissues and finally establish a new metastatic tumor in distant parts of the body. Although a tumor was originally thought to include only cancer cells, it is now known that cancer cells comprise only a part of what is known as the tumor microenvironment (TME) [1]. In fact, the TME is highly heterogeneous among patients, as although the main components are present in all tumors, the exact amount may differ significantly between tumors. Thus, various tumors may differ in the content of the TME, which may include varying amounts and compositions of extracellular matrix (ECM), the presence of immune cells [T and B lymphocytes, dendritic cells, natural killer cells, neutrophils, myeloid-derived suppressor cells (MDSC), and tumor-associated macrophages (TAMs)], cells of the vasculature (pericytes and endothelial cells), as well as stromal cells, such as fibroblasts and cancer-associated fibroblasts (CAFs) [2]. All TME components contribute to the cancer phenotype and are responsible for the observed inter-patient heterogeneity [2,3]. TME composition is therefore crucial for tumor progression and metastasis, while the amount of ECM and the resulting tumor stiffness have also been associated with increased tumor invasiveness and metastasis as well as therapy resistance [3].

Although extensive research has been conducted on cancer biology and the discovery of novel therapeutic approaches, effective cancer treatment is often a true challenge, posing more problems than offering a cure [4]. More specifically, conventional therapies such as chemotherapy and radiotherapy are usually accompanied by substantial toxic effects that affect the patients’ quality of life [5,6]. Moreover, despite the promising results of immunotherapy, this treatment modality can also be challenging, as its efficacy cannot be predicted, while developing resistance is also quite common. Thus, more research is imperative to develop more targeted and less toxic anti-cancer therapies or enhance the combination of existing therapies and reduce their side effects.

Among the novel anti-cancer strategies, photodynamic therapy (PDT) is gaining ground as an attractive therapeutic approach that could be easily combined with other therapies to enhance therapeutic outcome. PDT combines the use of a photosensitizer (PS) drug, a light source, primarily laser, and oxygen, and it can therefore be combined both with classical therapies, such as chemotherapy and radiotherapy, and with novel therapeutic approaches, such as TME-targeting therapies that aim to disrupt its pro-tumorigenic properties, and include antiangiogenic agents, ECM-modulating treatments, and immune-targeting strategies [3], as well as inhibitors of hypoxia-inducible factor-1 (HIF-1) [2]. In this review, we briefly present the key characteristics of PDT and its principles and highlight the effects of combining PDT with other therapeutic approaches, giving emphasis to the emerging TME-targeting therapies.

We, thus, explore the potential of PDT in combination with other cancer therapies, with a primary focus on TME-targeting strategies. This review aims to evaluate how these combination approaches enhance PDT efficacy, mitigate its limitations, and improve overall cancer treatment outcomes, highlighting promising avenues for future clinical application.

### 1.2. An Overview of Photodynamic Therapy (PDT)

**Figure 1:**
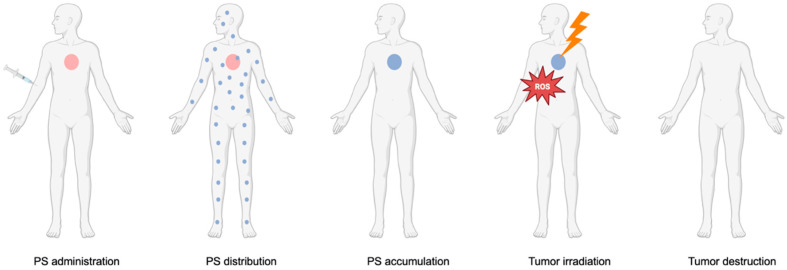
**Application of PDT in the clinical setting.** The PS is administered to the patient and distributed systemically. Once it accumulates in the tumor, the targeted area is irradiated with a light source, leading to ROS production and subsequent tumor destruction.

PDT is a non-invasive treatment that relies on the use of non-toxic components to generate reactive oxygen species (ROS) that are cytotoxic to cancer cells. Specifically, in PDT, there is interaction between a PS, a light source, and oxygen, which individually are non-toxic, to generate ROS [7]. Since each component is non-toxic on its own, PDT provides a targeted therapeutic effect with minimal systemic toxicity. The PS, a light-activated drug, can be administered orally, intravenously, or locally. During the drug-to-light interval (DLI), it selectively accumulates in tumor tissues, allowing precise activation when exposed to light of a specific wavelength [8]. Figure 1 shows the general concept of the application of PDT in the clinical setting.

While the exact mechanisms for the accumulation of PSs in the tumor tissues remain unclear, several factors have been proposed to contribute to the selective uptake by tumor sites [9]. One of these factors is the enhanced permeability and retention (EPR) effect that is characteristic of the TME, where blood vessels appear leaky with poor organization, resulting in easier PS accumulation than in normal tissues, where the vasculature is well organized and non-leaky. The EPR effect is accompanied by defective lymphatic drainage in tumors that increases PS retention at the tumor site [10]. PSs tend to bind to low-density lipoprotein (LDL) receptors, so another factor that affects the selective accumulation of PSs could be the increased expression of LDL receptors present in various cancer types [9].

Interestingly, compared to conventional cancer treatments such as chemotherapy and radiotherapy, PDT offers several advantages. More specifically, it is minimally invasive, does not require overnight hospitalization of the patient, and has fewer systemic side effects due to its localized nature [8]. Additionally, PDT can be applied repeatedly without significant toxicity, making it a viable option for long-term cancer management. Beyond its direct cytotoxic effects, PDT can stimulate immune responses and disrupt pathways involved in drug resistance, further enhancing its therapeutic potential [11]. Moreover, because PS activation occurs only in the presence of light, systemic toxicity remains low [12]. Finally, PDT has also been associated with impressive cosmetic outcomes, particularly in surface/skin cancers [8].

Despite these advantages, PDT also has certain limitations in its application. First, the depth of light penetration is restricted, making it less effective for treating deep-seated tumors. Another limitation is that PDT cannot be used for treating large tumors because light cannot penetrate deeply into biological tissues, while singlet oxygen, which is produced from the PDT reaction, has a short half-life, so PDT cannot be applied in large tissue areas. Moreover, since PDT is a localized treatment, it cannot be applied to cancer of advanced stage that has already metastasized, so it can only be used for treating regional malignancies and lesions of precancerous stage [13]. Finally, PDT has been associated with skin photosensitivity in patients after treatment [8], especially with certain PSs of the so-called first-generation PSs, as patients had to avoid light exposure, even from the sun, with clear consequences on patients’ life quality.

To address these limitations, current research efforts are focusing not only on developing better PSs and appropriate light sources but also on combining PDT with other cancer therapies, including chemotherapy, radiotherapy, and immunotherapy. In fact, since PDT targets different cellular mechanisms from chemotherapy and radiotherapy, the combined therapy can enhance treatment efficacy while reducing drug dose and associated side effects. Additionally, PDT has also been used in combination with antibody-based immunotherapies to improve tumor targeting [13]. Overall, these synergistic strategies have been shown to improve PDT’s therapeutic index, allowing for better tumor management and fewer side effects [14].

### 1.3. PDT’s Mechanism of Action

As mentioned above, one of the key components of PDT is the use of **PSs,** which, depending on their characteristics, can be categorized into three generations. The first generation emerged in the 19th century and consists of hematoporphyrin (Hp) and its derivatives (HpD). Hp has a heterotypical nature, and large doses were required to be effective as a cancer diagnostic tool, so it was purified further in the form of photofrin [7,15,16].

The second generation of PS overcame some serious disadvantages of the first generation, like short light wavelength and prolonged accumulation of the PS in normal tissues, and was based on chlorin and porphyrin structures that have a longer wavelength than those of the first generation [15]. These PSs have a high absorbance in the deep red, so they have an increased penetration of light, resulting in a more effective action towards the tumor [8]. One of the major disadvantages of second-generation PSs, however, is their low water solubility, which severely restricts their intravenous administration, presenting several challenges [16].

The third-generation PSs have a higher selectivity for regions of tumor tissues by conjugating or encapsulating PSs in carriers that can be transported to targeted tissues [15]. Antibodies, liposomes, carbohydrates, and proteins can be used as carriers to improve the chemical, physical, and therapeutic abilities of PSs [8]. Finally, third-generation PSs are designed to have better absorption of the ideal wavelength to improve tissue penetration [7]. A summary of the key characteristics of each PS generation is shown in Table 1 below.

Another key component of PDT is the selection of the appropriate **light source.** Various light sources have been employed so far based on the tumor’s location and the PS used, with lamps and lasers being the most commonly used. More recently, light-emitting diodes (LEDs) have emerged as a promising alternative for enhancing treatment flexibility and efficiency [17]. LEDs are suitable for PSs sharing the same wavelength and can be made portable. However, their large beam size and poor quality limit their use in endoscopic applications, as they cannot be paired with flexible optical fibers. For deeper tumors, lasers and LEDs are preferred due to their compatibility with flexible fibers. Natural sunlight can also be used for PDT, primarily in the treatment of skin lesions. When light penetrates the tumor tissue, it undergoes scattering, reflection, or absorption, which is affected by tissue type and light wavelength. Due to tissue heterogeneity, light absorption varies across different regions [7]. Longer wavelengths (600–850 nm), such as red light, penetrate tissues more effectively, while shorter wavelengths (<600 nm) have limited penetration and increase skin photosensitivity. Wavelengths beyond 850 nm cannot excite oxygen to its singlet state, leading to insufficient ROS production [8].

**Oxygen**, another component of PDT, is essential for ROS generation, while its availability within tumor tissues directly impacts treatment effectiveness. However, oxygen levels can vary significantly between different tumor types and even within regions of the same tumor [8]. In fact, hypoxia in tumor tissues can impair the tumor vasculature, ultimately affecting the delivery route of the PS. Thus, hypoxic tumor areas are considered obstacles to PDT efficacy and are sometimes resistant to PDT [7].

**Figure 2:**
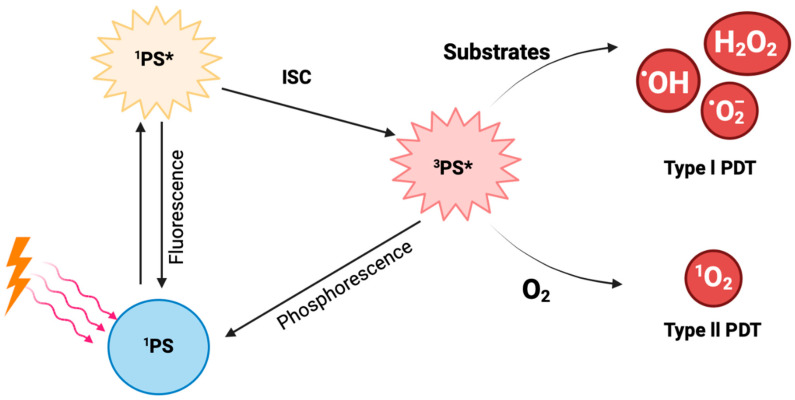
**Diagrammatic representation of the photodynamic reaction from the moment the PS absorbs photons from the light source to the generation of free radicals**.

The photodynamic reaction is initiated when photons from the light source are absorbed by the PS (Figure 2). This illumination transforms the ground state PS, which is called the singlet state (^1^PS), to its excited singlet state (^1^PS*) [18]. The excited singlet state lasts for a few nanoseconds, thus being very short-lived and unstable. Subsequently, and through intersystem crossing (ISC), the PS can transform into its triplet state, ^3^PS* [17]. This triplet state is long-lived and more stable than the singlet state, and it can undergo two types of reactions, namely Type l and Type ll. A Type l reaction occurs when the triplet state reacts with different substrates, yielding free radicals or radical ions. The reaction between the generated radicals and O_2_ results in the production of ROS like hydroxyl radical (HO^•^), hydrogen peroxide (H_2_O_2_), and superoxide anion (O2^−•^), which cause oxidative damage to the cells. In Type II reactions, the longer lifetime of the triplet state provides enough time for energy to be directly transferred to molecular oxygen (O_2_), leading to the production of singlet oxygen (^1^O_2_) [8].

Interestingly, the two reaction types can occur at the same time, although it is widely accepted that most PSs mainly use the Type II reaction [17]. The rate by which these two reactions occur depends on oxygen concentration, the PS, the substrate present, and the PS’s binding affinity to the substrate [8].

Most PDT treatments that are currently applied are based on Type II reactions, so a large amount of oxygen is required during treatment. Consequently, Type II PDT reactions have the potential to make hypoxia worse and lead to a less effective therapeutic outcome [19]. The exact role of oxygen in Type I PDT remains debated, but studies indicate that Type I PDT is more effective than Type II in hypoxic environments. A way to increase the presence of oxygen in tumor tissues is the use of hyperbaric oxygen (HBO) treatment, where patients are provided with pure oxygen in a pressurized chamber. This method increases oxygen availability and alleviates hypoxia in tumor tissues [20]. Another approach involves directly transporting oxygen to the tumor using carriers like hemoglobin and perfluorocarbons [8].

## 2. Combination of PDT with Other Therapies

In order to maximize the benefits from PDT treatment while overcoming current limitations, PDT is given in combination with other therapeutic approaches. More specifically, PDT has been used in combination with the following:

**A. Chemotherapy:** In chemotherapy, cytotoxic agents are administered to the patient, aiming to destroy the tumor [21]. Several studies have combined chemotherapeutic agents with PDT. Morita et al. (2020) investigated the safety and efficacy of combining PDT with chemotherapy [22]. The study demonstrated that combining bexarotene, used for cutaneous T-cell lymphoma treatment, with PDT resulted in a better therapeutic outcome compared to bexarotene monotherapy. Along the same line, Zhang et al. (2023) investigated a pH-responsive drug delivery system based on conjugated polymers (PFE-DOX-2) for effective synergistic chemotherapy combined with PDT for treating breast cancer [23]. Doxorubicin (DOX), a widely used chemotherapeutic agent, was attached to the polymers to prevent premature drug leakage and enhance tumor cell uptake. The combined treatment with PDT significantly reduced cell viability and increased necrosis, demonstrating the enhanced therapeutic potential of this approach. The combination of DOX with PDT was also used by Chilakamarthi et al. (2023), where low-dose DOX was combined with a porphyrin-based PS (P-nap) to treat colorectal cancer in mice [24]. The combination therapy, along with irradiation, led to significant tumor reduction by increasing the Bax/Bcl-xL ratio and p53 expression and promoting apoptosis. Additionally, the downregulation of Phosphoinositide 3-kinase (PI3K) and Cyclooxygenase 2 (COX-2) pathways contributed to tumor suppression and relapse prevention. Other chemotherapeutic agents have also been explored, such as ruthenium-based complexes used to generate PSs for colorectal cancer treatment. The ruthenium-enhanced drug internalization led to a higher number of cancer cells arrested in the S phase, while upon irradiation, the treated cell lines showed reduced viability, increased ROS production, and enhanced apoptosis [25].

**B. Immunotherapy:** In immunotherapy, the tumor is destroyed by stimulating distinct components of the patient’s immune system [26]. As immunotherapy emerges as a promising strategy for treating various cancer types, its combination with other therapeutic modalities, including PDT, has gathered significant scientific interest due to its potential to enhance treatment efficacy. One promising example is the combination of nanoparticles loaded with the PS drug Temoporfin and a PD-L1 blockade antibody, which has demonstrated the ability to inhibit primary tumor growth and delay the progression of distant tumors in colorectal cancer [27]. Since PDT often upregulates PD-L1 expression, allowing cancer cells to evade immune detection, the addition of a PD-L1 inhibitor effectively counteracts this mechanism by enhancing immune recognition and promoting apoptosis. This combination not only improved tumor suppression but also promoted long-term survival by significantly enhancing the activation and response of CD8+ T cells, a crucial component of the antitumor immune response [27]. PDT was also combined with an autophagy inhibitor (CQ) for treating colorectal cancer and resulted in the enhancement of the maturation of infiltrating CD8+ T cells and DCs [28]. Two other studies utilized the combination of PDT with monoclonal antibodies and its effectiveness as a cancer therapeutic. One of them used an immunotoxin (saporin) conjugated to cetuximab, an Epidermal Growth Factor Receptor (EGFR) inhibitor, causing cell death, inhibiting recurrence, and increasing antitumor cytotoxicity for the treatment of lung cancer [29]. Similarly, Yamashita et al. (2023) showed that the use of monoclonal antibodies (panitumumab or trastuzumab) along with PDT induces cell death 24 h after radiation and binds specifically to cancer cells, sparing the neighboring tissues from any undesired effects of the treatment [30]. Additionally, the combination of PDT with sorafenib, a multi-kinase inhibitor known to induce necrosis, has been explored. When delivered via nanoparticles that enhance drug internalization, this combination demonstrated an improved antitumor effect in thyroid and breast cancers. In the study, nanoparticles loaded with the PS drug chlorin e6 and sorafenib significantly reduced cell viability and tumor volume. The treatment also promoted increased recruitment of cytotoxic T cells and enhanced inflammation, contributing to tumor suppression in both primary and distant lesions [31].

Another way of combining immunotherapy and PDT is with the use of vaccines. Cancer cells treated with PDT were used to prime dendritic cells (DC) to create vaccines. This method was shown to enhance the response of cytotoxic T cells (CTL) against tumors [32], increase survival, decrease tumor size [33], and increase the number of CD8+ T cells in the lymph nodes surrounding the tumor area [34]. PDT-treated cancer cells were used to create vaccines by another team that used a stimulant of immune activities (GC) along with the vaccine to treat tumors. The results suggest that this combination has an additive therapeutic effect as the number of dead cells almost doubled and the progression of the tumor was delayed [35]. Lastly, the combination of PDT with another vaccine type (FlaB-Vax) was shown to extend survival, suppress both primary and secondary tumors, and induce systemic CD8+ T-cell tumor-specific responses [36].

However, although combining PDT with chemotherapy, radiotherapy, or immunotherapy has shown significant promise, additional combined therapies have also been suggested to include other targeted treatments. For instance, the combination of PDT with vismodegib, a drug targeting the Sonic Hedgehog signaling pathway, has been explored in basal-cell carcinoma patients. This combination resulted in excellent cosmetic outcomes with minimal adverse effects on patients’ daily activities [37].

**C. Radiotherapy:** In radiotherapy, ionizing radiation is used to destroy cancer cells, inhibiting their growth [38]. Recent years have seen growing scientific interest in combining PDT with radiotherapy. To the best of our knowledge, only a couple of research articles present the combination of PDT with radiotherapy. For instance, Bulin et al. (2019) combined PDT with X-rays to treat colorectal cancer [39]. Cells treated with both modalities showed significantly higher necrosis compared to those treated with either PDT or radiotherapy alone. The combination also led to a greater reduction in tumor size, inhibiting cancer cell growth, while PDT induced necrosis, creating a synergistic therapeutic effect when applied together. Another study by Mayahi et al. (2019) showed that PDT followed by X-ray radiation increased cell death in MCF-7 cells and significantly decreased cell survival [40]. Liu et al. (2021) used nanoparticles (RGD-PEG-PAA-MN@LM) for PDT combined with radiotherapy [41]. Their findings indicate that using these nanoparticles for PDT followed by X-ray irradiation can increase ROS production and decrease the tumor size. Lastly, the team of Zhou et al. (2023) developed a nanoplatform containing red blood cell-coated nanoparticles loaded with the PS Ce6 for PDT and radiotherapy combination [42]. This system enabled deep-tissue treatment and triggered antitumor immune responses, effectively suppressing tumor growth and metastasis.

**D. As part of triple-combination strategies:** Several studies have compared different therapeutic combinations or explored triple-combination approaches. One study examined a triple combination of PDT with radiotherapy and immunotherapy. PDT-killed SCC7 cells were used to prime dendritic cells (DC) to create vaccines. Mice with squamous-cell carcinoma (SCC7) tumors treated with the DC-based vaccine showed improved survival rates and delayed tumor growth compared to PDT alone. In fact, when irradiation was performed around the time of vaccination, tumor growth inhibition was further enhanced compared to pre-vaccination irradiation [43]. Another study demonstrated that combining PDT with chemotherapy and immunotherapy was also effective in inhibiting tumor growth in both primary and metastatic lung lesions of triple-negative breast cancer (TNBC). The combination therapy utilized a prodrug capable of releasing DOX and caspase-3, which promoted apoptosis. When paired with immune checkpoint inhibition (anti-PD-L1), it further enhanced the maturation of dendritic cells and the activation of cytotoxic T lymphocytes [44]. Furthermore, a study by Kim et al. 2021 investigated the combination of PDT, a PD-1/PD-L1 immune checkpoint inhibitor, and the rho-kinase (ROCK) inhibitor ripasudil, resulting in significant tumor growth inhibition in both primary and metastatic lesions [45].

A summary of the studies that combined PDT with chemotherapy, radiotherapy, or immunotherapy is presented in Table 2 below.

## 3. PDT and TME-Targeted Therapies

### 3.1. TME-Targeting Therapies

A novel anti-cancer therapeutic approach that is gaining ground is TME-targeting therapy.

TME-targeting therapy includes various strategies, all of which target different TME components.

(a) One strategy is to try to reduce the ECM content of the tumor that usually affects drug delivery by compressing blood vessels. In this strategy, ECM components such as collagen are specifically targeted with angiotensin ll receptor agonists such as Losartan, which has been shown to reduce collagen l secretion [46].
(b) Another strategy is using inhibitors of HIF-1to tackle hypoxia present in the TME, such as Topotecan, a topoisomerase 1 inhibitor, and Digoxin, which targets HIG1A [2,47].
(c) A third strategy is to target pericytes and endothelial cells to prevent neovascularization using Anti-Vascular Endothelial Growth Factor (VEFG) antibodies, such as Bevacizumab, and mTOR inhibitors to decrease angiogenesis [48,49]. In fact, Bevacizumab, a humanized monoclonal antibody for VEGFA, was the first antiangiogenic therapy to receive FDA approval in 2004 for treating the TME of different cancers such as glioblastoma and cervical cancer [3].
(d) The diverse immune composition within the TME can also be targeted through various routes. One approach is decreasing TAM and myeloid-derived suppressive cell (MDSC) infiltration by using small-molecule inhibitors or neutralizing antibodies against CDF-1 or CD204 [50,51,52]. IL-1 receptor antagonists, like Anakinra and Canakinumab, can be used for targeting chronic inflammation, which is closely related to carcinogenesis [53,54].
(e) Finally, other targetable components of the TME are CAFs, with many strategies currently under investigation to modulate their activity, such as the targeting of fibroblast activation protein α (FAP) with an interleukin-2 variant (RO6874281), which targets FAP [2].

**Figure 3:**
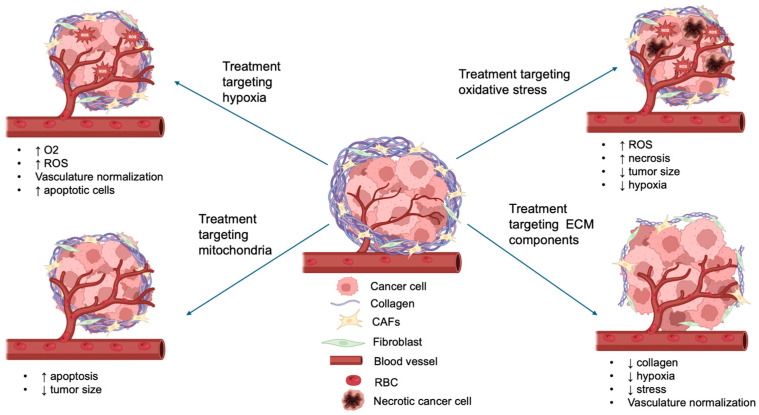
**Schematic overview of the TME components targeted by different therapeutic strategies in combination with PDT.** The diagram highlights representative approaches against hypoxia, oxidative stress, extracellular matrix (ECM) components, and mitochondria, illustrating how these interventions enhance PDT efficacy (↑ indicates increase; ↓ indicates decrease).

Thus, TME-targeting strategies encompass a wide range of approaches that interfere with distinct components of the tumor environment. These include reducing extracellular matrix density to improve vascular perfusion; alleviating hypoxia through HIF-1 inhibition or oxygen delivery; suppressing angiogenesis by targeting VEGF signaling; modulating immune infiltration, such as TAMs and MDSCs; and regulating CAF activity. Together, these strategies reshape the tumor milieu, enhance drug delivery, and create conditions that support the efficacy of complementary treatments such as PDT (Figure 3).

Notably, TME-targeted therapies offer distinct advantages compared to other targeted strategies. Unlike conventional targeted therapies that act on single molecular drivers, TME-targeted approaches modulate the entire tumor ecosystem. By altering various TME components, these therapies can improve drug penetration and enhance the efficacy of co-administered treatments. Importantly, they also address tumor heterogeneity, which often limits the success of therapies directed against single oncogenic pathways. This broader mode of action makes TME-targeted therapy a highly complementary partner for PDT, providing synergistic effects that are less likely to be overcome by resistance mechanisms.

### 3.2. Combination of TME-Targeting Therapies

#### 3.2.1. Targeting Hypoxia and TME Vasculature

With the growing interest in developing novel therapeutics that target the TME, combining TME-targeting therapies with PDT is a rather appealing approach. In fact, there have been several studies combining PDT with various TME-targeting therapies. Specifically, Lin et al. (2022) [55] used polymer-encapsulated carbonized hemin nanoparticles (P-CHNPs) as the PS drug to tackle the oxidative stress generated in the TME. Hemin is a Fe(III)-containing proto-porphyrin IX that can catalyze oxidation reactions and promote the production of ROS. Cells treated with P-CHNPs and irradiated exhibited an increased extent of necrosis, and the size of the tumors significantly decreased. The light from the irradiation during PDT further enhanced the conversion of H_2_O_2_ to HO^•^ or O_2,_ resulting in decreased hypoxia and enhancing the anti-cancer effect. Interestingly, the regulation of oxidative stress was more targeted to the TME than the normal surrounding tissues, and the tumor size was significantly reduced in contrast to when PDT is used alone, where the tumor growth is suppressed, but a decrease in tumor size is hard to achieve.

Moreover, several studies have highlighted the impact of using nanoparticles to reduce hypoxia, which is a fundamental characteristic of the TME. More specifically, conjugated nanoparticles linked to hemoglobin (Hb) and encapsulated into liposomes (Hb–NPs@liposome, which was the PS) were used by Jiang et al. (2019) [56] to increase the oxygen production in the TME. No external light source was used because luminol and H_2_O_2_ were added, and Hb can activate luminol to emit blue light. The results showed that the hemoglobin nanoparticles can be internalized by the cancer cells when the liposome interacts with them. As a result, ROS production was increased, and the cell viability of cervical cancer cells was reduced [56]. Lee et al. (2022) [57] used nanoparticles loaded with a chemotherapeutic drug that targets microtubules, named paclitaxel (PTX), and the PS drug chlorin e6 (Ce6) for treating TME hypoxia and showed a reduction in cell viability and inhibition in tumor growth via activation of the p53 signaling pathway. The nanoparticles were created by using paclitaxel loaded in human serum albumin (HSA) conjugated with Azo, which is a linker that reacts to hypoxia. These nanoparticles also contained Ce6 and RGD derivatives (RP/CA/PHNPs). The Azo group facilitated the distribution and release of Ce6, increasing the efficacy of PDT and ROS production. This increase in the production of ROS resulted in the activation of the p53 signaling pathway and the decrease in tumor development [57].

Similarly, Zhang et al. (2022) [58] aimed to reduce hypoxia by using nanoparticles (AFZDA) to deliver the PS to the tumor site. These nanoparticles contained gold, zinc, iron, DOX, and angiogenin-2, which is thought to promote the formation of blood vessels, which helps in the normalization of the tumor vasculature. The results indicated that by using these nanoparticles, there was increased oxygen and drug delivery to the tumor site by normalizing the tumor vasculature through changes in their structure and morphology, ultimately resulting in extending the life of the treated mice [58]. Zhu et al. (2020) [59] used nanovesicles combined with the glucocorticosteroid dexamethasone to alleviate hypoxia by vascular normalization. By combining the nanovesicles with dexamethasone and laser irradiation, tumor growth inhibition was achieved, and ROS production was increased due to successful vascular normalization with no side effects [59]. Another study also used dexamethasone (Dex) to treat colon cancer in mice. When PDT was applied, survival pathways such as HIF-1α and NF-κB were found to be activated, and Dex was reported to inhibit these pathways. In this study, a porphyrin-based PS containing methoxy naphthalene (P-nap) was used. The result of using Dex along with PDT was an increase in the ratio of the pro-apoptotic Bax over the anti-apoptotic Bcl-xL towards apoptosis, as well as an increase in the expression of tumor suppressor p53. Moreover, a downregulation in HIF-1α and c-Myc was observed, which is regulated by NF-κB, enhancing tumor suppression and increasing the survival rate [60].

Another example is the use of different kinds of nanoparticles (PFOB@IMHNPs) that can deliver oxygen, PS (mTHPC), and a photothermal agent (IR780) into the site of the tumor. The results of Kv et al. (2022) [61] showed that after laser irradiation, PFOB@IMHNPs suppressed tumors and relieved hypoxia in the TME, resulting in full inhibition of tumor growth both in vitro and in vivo. This was achieved by increasing ROS production and temperature in the TME through the PS and the photothermal agent [61]. Liang et al. (2020) [62] used a nanoplatform (PDA-Pt-CD@RuFc) that could produce oxygen and ROS. After the samples were incubated with the nanoplatform and irradiated with a laser, the number of apoptotic cells increased dramatically, and inflammatory TME markers as well as genes related to hypoxia were downregulated [62].

Notably, the combination of PDT with nanoparticles and chemotherapeutic agents was explored in two studies. Yang et al. (2022) [63] used a nanoplatform (Fe_3_O_4_/Au NCs@LCPAA-TPP) responsive to TME that contained gold nanoclusters as PS along with hydrogels, ferric oxide (Fe_3_O_4_), and mitochondria-localized triphenylphosphine derivatives (TPP). Irradiation of the nanoplatform with a laser resulted in tumor volume reduction as the absorption of its contents was broadened by Fe_3_O_4_, and consequently, apoptosis of the tumor cells was triggered [63].

#### 3.2.2. Targeting Mitochondria and Other ECM Components

A nanosystem (MND-IR@RESV) that reacts in the presence of ROS and accumulates in mitochondria was created by Peng et al. (2023) [64]. When stimulated by an NIR laser, the nanosystem selectively accumulated in the tumor and improved the cancer cells’ mortality as the volume of the tumor was decreased and apoptosis was induced. The nanosystem had minor side effects, and its toxicity was low in normal tissues [64]. Finally, the team of Wang et al. (2020) [65] used an anti-fibrotic drug loaded into a polymer to target components of the ECM, like collagen l and hyaluronic acid. The results indicated that this nanosystem can selectively accumulate in the TME, and the amount of collagen l and hyaluronic acid decreased dramatically. Therefore, the solid stress of the tumor was decreased due to the destruction of the ECM, and hypoxia was reduced as the vasculature of the tumor became less leaky. Moreover, the nanosystem had negligible systemic toxicity in mice as there was no fluctuation in their weight, and no metastatic lesions were observed [65].

Table 3 below summarizes the studies discussed above that combined PDT with TME-targeting therapies.

## 4. Discussion

PDT has been studied extensively for cancer treatment over the past years, as it offers some important advantages, including localized action, minimal side effects, repeatability, and excellent cosmetic outcomes. However, despite its potential, it remains underutilized in clinical settings. In this review, we studied the current literature on the combination of PDT with conventional cancer therapies (chemotherapy, radiotherapy, and immunotherapy) as well as with the novel TME-targeting strategies to enhance overall therapeutic efficacy.

The findings demonstrate that combining PDT with these treatments improves outcomes by enhancing therapeutic effects while reducing the adverse effects associated with conventional therapies. Such combinations have the potential to overcome limitations in standard treatments and optimize the benefits of PDT, making it a promising strategy for future cancer therapeutics.

The combination of PDT with chemotherapy has proven more effective than chemotherapy alone, as demonstrated by studies involving cancer patients, mice, and cell lines [22,23,25,44]. Similarly, combining PDT with radiotherapy showed enhanced tumor suppression in mice, with further improvements observed when DC vaccination was incorporated into the treatment scheme [39,43]. The combination of PDT with immunotherapy also showed promising results across various cancer types, with half of the reviewed studies reporting therapeutic effects not only in primary tumors but also in distant lesions [27,28,29,31,36,45].

Moreover, we gave emphasis to studies that combined PDT with TME-targeting therapies, as the unique characteristics of the TME pose significant obstacles to PDT’s effectiveness. Targeting key features such as hypoxia can help overcome these barriers and enhance PDT’s therapeutic potential. The results of studies combining PDT with TME-targeting therapies demonstrated that targeting specific components of the TME can effectively enhance tumor destruction and improve therapeutic outcomes. Using therapeutic approaches that increase the production of ROS, essential for the photodynamic reaction, or normalizing the vasculature present in the tumor site are some of the methods proven to have the ability to increase the cytotoxicity towards cancer cells and, as a result, inhibit the growth of the tumor and enhance the photodynamic effect. Using nanoparticle systems to deliver the PS inside cells results in improvements of targeted delivery to specific cells or tissues, so the accumulation and uptake of the drug by the target cells is enhanced, while the rest of the tissues remain relatively or even completely unaffected.

**Figure 4:**
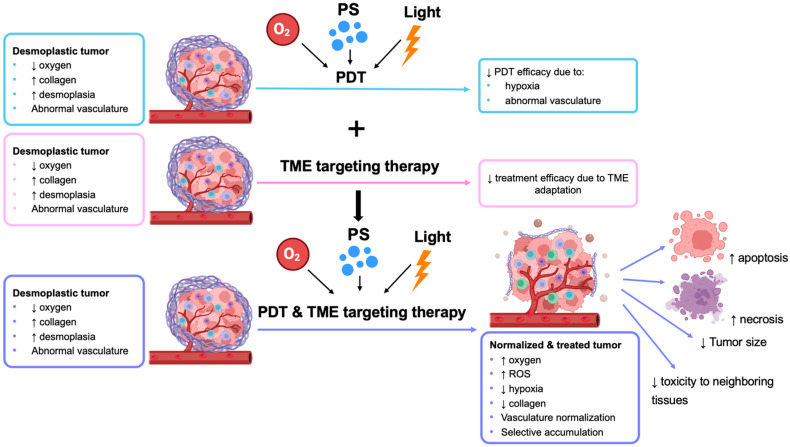
**PDT combined with TME-targeting therapy.** The figure shows the limitations of PDT monotherapy, TME targeting therapy alone, and the advantages of combining PDT with TME-targeting therapy (↑ indicates increase; ↓ indicates decrease).

According to the findings discussed above, a summary of studies using a combination of PDT and TME-targeting therapies is presented in Figure 4 below.

Notably, though, further research is needed for these methods to be used in the clinical setting, as, so far, all the evidence of their effectiveness is derived from experiments on cell lines and mouse models.

Despite the promising results of PDT combined with other therapies in preclinical settings, the path to clinical success remains uncertain and challenging. While animal models and cell-based experiments demonstrate significant tumor regression and reduced side effects, translating these findings into human trials has proven inconsistent [66]. Many preclinical studies fail to account for the complexity and variability of human tumors, including patient-specific factors such as genetic heterogeneity, immune responses, and tumor microenvironment dynamics. As a result, what works in controlled laboratory conditions often falls short in real-world applications, raising concerns about the robustness of current combination approaches. For example, modulating hypoxia may unintentionally lead to adaptive tumor responses, such as enhanced blood vessel formation or metabolic changes, which can reduce the effectiveness of the treatment. Moreover, nanoparticle-based delivery systems, while theoretically improving PS uptake and specificity, face obstacles related to systemic toxicity, immune clearance, and insufficient drug penetration in solid tumors. Most of the studies discussed relied on mice or cell models, highlighting the need for further research for a successful transition to clinical application [23,24,25,27,28,29,30,31,32,33,34,35,39,43,44,45]. Additional studies and clinical trials are essential to evaluate the effectiveness of these combination therapies in patients, enabling their potential integration into clinical practice and enhancing cancer treatment outcomes.

The analysis of the reviewed studies highlights the significant potential of combining PDT with other cancer treatments or TME-targeting therapies for treating various cancer types. Actually, the combination of PDT and TME-targeting therapies has demonstrated remarkable therapeutic effects in cell lines and animal models, suggesting promising prospects for future clinical application [55,56,57,58,59,60,61,62,63,64,65]. Further research should focus on deepening the understanding of TME dynamics and exploring innovative ways to enhance PDT’s efficacy, such as targeting CAFs, reprogramming immune cells within the TME, or leveraging nanotechnology for more precise and effective treatment delivery.

The strategies depicted in Figure 3 offer certain therapeutic advantages, but they also bear certain limitations.

For instance, treatments targeting hypoxia have shown promise as they increase oxygen, enhance ROS production, and also increase the number of apoptotic cells, but at the same time, the majority of these therapies have not reached late-stage clinical trials either due to complications pertaining to tumor heterogeneity or induced resistance [67]. Along the same line, oxidative stress treatments have the ability to elevate ROS production and decrease hypoxia and tumor size, but they also lead to necrosis, which can be toxic for healthy neighboring cells.

Treatments that target mitochondria seem to be more promising as they lead to increased antitumor activity both in vitro and in vivo with minimal toxicity and low side effects in normal tissues. Similarly, treatments targeting components of the extracellular matrix decompress blood vessels, enhancing drug delivery and promoting ROS generation while also enhancing the PDT effect. Moreover, this modality has been shown to decrease the metastatic ability of the cells with low systemic side effects [65]. To address the above-mentioned limitations, future research could focus on combining different treatments, such as HIF inhibitors and vasculature normalization, to increase treatment efficacy. Future research should also explore developing medical equipment that can easily deliver light to deep tissues so the effect of each of these therapies, as well as the efficacy of PDT, is maximized.

While the majority of studies report positive outcomes for PDT-based combination therapies, a deeper comparative analysis reveals several important points. Strategies targeting hypoxia and vascular normalization consistently show strong preclinical efficacy, but conflicting results highlight the risk of adaptive responses such as compensatory angiogenesis, which may reduce long-term benefits. Similarly, nanoparticle-based delivery platforms demonstrate enhanced PS accumulation and ROS production, yet they face challenges with systemic toxicity, immune clearance, and poor scalability, raising concerns about their translational feasibility. By contrast, immune-targeting approaches, particularly those that combine PDT with checkpoint inhibitors, have shown the most durable and systemic antitumor effects, although variability in response rates across tumor models underscores the need for better patient stratification. Overall, while TME-directed approaches hold great promise, those integrating PDT with immunotherapy appear most likely to advance toward clinical success, whereas nanoparticle-heavy strategies remain limited by manufacturing and regulatory hurdles. A clearer understanding of these limitations and conflicts will be essential for prioritizing future research directions and guiding rational clinical trial design.

The clinical translation of PDT, particularly in combination with TME-targeting strategies, encounters several barriers. Regulatory challenges arise from the diversity of approval pathways and the complexity of evaluating PDT as a drug–device combination [68]. Different regions’ jurisdictions, including the United States, Europe, and Japan, apply inconsistent standards for premarket evaluation, registration procedures, and optimization of therapeutic parameters [69]. For instance, PS sensitizers, such as Photofrin, BPD-MA, ALA, and MAL, have received approval for distinct cancer indications depending on regional criteria, yet significant discrepancies remain in drug–light interval assessment and light power density optimization between U.S. and Japanese studies [70]. In addition, inconsistent trial registration and incomplete evidence reporting reduce transparency and hinder post-market surveillance [71]. Beyond regulatory issues, manufacturing and scalability present further challenges, particularly for nanoparticle-based PDT systems. PS-loaded nanoplatforms frequently face hydrophobicity, solubility, and aggregation problems, while complex synthesis pathways limit reproducibility and large-scale production [72]. The absence of standardized long-term toxicity evaluations remains a significant obstacle, as noted in preclinical studies on nanostructure-based systems [73,74]. Addressing these challenges is critical for the safe and effective advancement of PDT–TME approaches.

Despite these barriers, PDT is making progress in clinical evaluation. Several trials are actively investigating its role in immunomodulation, chemotherapy enhancement, and TME modulation. For example, a clinical trial in basal-cell carcinoma is studying how PDT alters the immune microenvironment [75]. In lung cancer, photoimmunotherapy with ASP-1929 combined with cemiplimab is being tested for refractory, inoperable, and metastatic non-small-cell lung cancer [76]. In pancreatic cancer, verteporfin-mediated photoradiation is being combined with pembrolizumab and chemotherapy to evaluate local and systemic immune responses [77]. In cholangiocarcinomas, PDT is under evaluation as both a comparator to radiofrequency ablation and as a neoadjuvant approach to improve resectability [78,79]. Other active studies include glioblastoma [80], recurrent prostate cancer [81], intermediate-risk prostate cancer [82], recurrent superficial esophageal carcinoma [83], and extrahepatic cholangiocarcinoma [78,84]. Collectively, these studies illustrate a growing clinical interest in PDT, particularly when integrated with immunotherapy or TME-targeted strategies, and highlight the need for harmonized regulatory frameworks and robust manufacturing solutions to facilitate broader clinical adoption.

## 5. Conclusions

This literature review highlights the usefulness of combining PDT with chemotherapy, radiotherapy, and immunotherapy, producing a synergistic therapeutic effect across various cancer types. The main focus, however, was on the combination of PDT with TME-targeting therapies, which address critical barriers to treatment success, such as increased ECM deposition, hypoxia, abnormal vasculature, and immune suppression. By targeting these features, TME-focused combinations enhance the effectiveness of PDT, leading to improved tumor destruction, increased apoptosis, and reduced resistance to treatment. Additionally, innovative delivery systems, such as nanoparticles, improve PS uptake, boost ROS production, and minimize off-target effects. These promising preclinical outcomes underscore the need for further research and clinical trials to bridge the gap between research and clinical application. With continued development, PDT-TME combination therapies hold the potential to overcome current limitations and redefine cancer treatment strategies.

Future research should prioritize the integration of PDT with TME-targeted strategies that specifically enhance antitumor immunity. Approaches that remodel the ECM, normalize tumor vasculature, alleviate hypoxia, or reprogram immunosuppressive cell populations can create a more favorable environment for immunotherapy. Combining these interventions with PDT has the potential to increase immune infiltration, improve antigen presentation, and promote durable systemic responses. Parallel advances in imaging-guided PDT could enable real-time monitoring of treatment efficacy and PS distribution, while tumor profiling may support personalized selection of photosensitizers and delivery platforms. These directions are expected to accelerate the translation of PDT–TME therapies into clinical practice and maximize their therapeutic potential in cancer treatment.

## Figures and Tables

**Table 1:** Summary of the key characteristics of PDT.

PS Generation	Key Characteristics	Representative PS
1st	Poor tissue penetration Skin hypersensitivity	Porphyrin Hematoporphyrin
2nd	Higher chemical purity Better tissue penetration Poor water solubility	Benzoporphyrin 5-Aminolevulinic acid
3rd	Higher tissue selectivity Lower required dose	Monoclonal antibodies conjugated with PS

**Table 2:** Summary of studies combining PDT with chemotherapy, radiotherapy, or immunotherapy.

Reference	Type of Cancer	Type of Treatment	PDT Light Source	PS Type	Sample	Result
[22]	Cutaneous T-cell lymphoma in humans	Chemotherapy (bexarotene 300 mg/m^2^)	PUVA or UVB (0.5–4.0 J/cm^2^ for UV-A, 0.5 2.0 J/cm^2^ for UV-B)	Psoralen	CTCL patients	Higher response rate than monotherapy with chemotherapy
[23]	Human breast cancer	Chemotherapy (Doxorubicin)	White light (25 mW cm^−2^ for 30 min)	PFE-DOX-2	Human breast cancer cells	Powerful synergistic chemo-/PDT therapeutic effect
[24]	Colon cancer in mice	Chemotherapy (Doxorubicin)	1200 W lamp (20 J cm^−2^, 50 mW cm^−2^, 600–720 nm)	P-nap 30 μM for 24 h	Colon cancer cell line	Tumor regression, relapse prevention
[25]	Colorectal in humans	Chemotherapy (ruthenium-based complexes)	Lamp (630–660 nm, 75 J/cm^2^)	2H-TPyP-arene-Ru and Zn-TPyP-arene-Ru	CRC cell lines	Cell viability decreased, increased rate of apoptosis
[27]	Colorectal in mice	Immunotherapy (anti-PD-L1 antibody, 100 μg i.v. injection)	NIR laser (660 nm, 0.72 J/cm^2^ for 5 min)	Temoporfin (0.3 mg/kg)	Subcutaneous CT26 tumors in BALB/c mice	Combination inhibits growth of tumors
[28]	Colorectal in mice and humans	Immunotherapy (Chloroquine, 25 mg/kg)	Laser (635 nm, 40 J/cm^2^, 3 min)	5-aminolevulinic acid (50 mg/kg)	Various cell lines	Combination results in tumor suppression
[29]	Lung cancer in humans	Immunotherapy (immunotoxin containing saporin and cetuximab 0.001–8 nM)	Laser (664 nm)	Mono-L-aspartyl chlorin e6 (NPe6) (0.1, 1, 3 mg/kg)	Human lung cancer cells	Enhancement of antitumor effect
[30]	Human breast ductal carcinoma, human epidermal carcinoma	Immunotherapy (panitumumab or trastuzumab 6.8 nmol)	Laser (690 nm, 50 mW/cm^2^)	mAb-IR700/talaporfin Dose not available	Various cell lines	Combination provides additive treatment effect
[31]	Human thyroid and breast cancer in mice	Immunotherapy (sorafenib 0.62 mg/kg)	Laser (660 nm, 0.05 W/cm^2^ for 120 min)	Chlorin (0.65 mg/kg)	Subcutaneous K1 tumor in BALB/c mice	Strong antitumor immune response induction
[32]	Skin squamous-cell carcinoma in mice	Immunotherapy (DC vaccine)	LED (630 nm, 10 mW/cm^2^, 0.5 J/cm^2^)	5-aminolevulinic acid (0.5 mM for 5 h)	Subcutaneous tumors in SKH-1 mice	ALA-PDT DC vaccine induces systemic antitumor responses
[33]	Peritoneal mesothelioma in mice	Immunotherapy (DC vaccines 2 × 10^6^ DC in 100 μL PBS i.p.)	Daylight LED (2.55 mW/cm^2^) for 1 h, 9.18 J/cm^2^)	OR141 Dose not available	Mesothelioma cell lines and in situ Ab-1 Luc tumors in BALB/CByJ mice	Induction of strong immune response against mesothelioma
[34]	Glioma in mice	Immunotherapy (DC vaccines loaded with glioma cells undergoing ICD by PDT)	Not available (20 J/cm^2^)	Photosens	Subcutaneous and in situ GL261 tumors in C57BL/6 J mice	Combination is effective in treating glioma
[35]	Squamous-cell carcinoma in mice	Immunotherapy (PDT-generated cancer vaccine, 2 × 10^7^ cells/mouse injected peritumorally)	Lamp (665 ± 10 nm, 1 J/cm^2^, 30 mW/ cm^2^)	Chlorin e6 (1 μg/mL)	Subcutaneous SCCVII tumors in C3H/HeN mice	Immunotherapy is an adjunct to PDT
[36]	Melanoma in mice	Immunotherapy (Flab-Vax vaccine)	Laser (674 nm, 200 mW/cm^2^ for 15 min)	pheophorbide A (PhA) (5 mg/kg)	In situ B16-F10 tumors in C57BL/6 J mice	Combination suppresses tumors
[37]	Multiple basal-cell carcinomas in humans	Immunotherapy (vismodegib 150 mg by mouth every day for 3 months)	LED lamp (630 nm, 75 J/cm^2^ for 20–24 min)	5-aminolevulinic acid Dose not available	BCC patients	Combination results in effective treatment of BCC
[39]	Pancreatic in humans	Radiotherapy (X-rays 2.75 Gy/min)	NIR laser (690 nm, 150 mW/cm^2^)	benzoporphyrin-derivative (0.25 μmol/L)	PanCan cell lines	Restriction of tumor growth and increased necrosis
[40]	Breast in mice	Radiotherapy (X-rays 2 Gy/min)	Laser (660 nm, 150 mW, 15.7 mW/cm^2^, 1.8 and 2.8 J/cm^2^ for 120 and 180 s)	GaPcCl (50–100 μg/mL)	MCF-7 cells	Decreased cell survival Increased apoptosis
[41]	Human liver cancer in mice	Radiotherapy (X-rays)	NIR (808 nm, 2.0 W·cm^−2^ for 5 min	RGD-PEG-PAA-MN@LM (100 μL)	Subcutaneous HepG2 tumors in BALB/c mice	Increased ROS production Tumor size decreased
[42]	Breast in mice	Radiotherapy (X-rays)	NIR light (10 min, 1 min interval, 0.25 W/cm^−2^)	RBC/Ce6/UCNPs (0.1 mmol Gd per kg body weight	Mice with 4T1 tumors	Increased ROS production Enhancement of antitumor immune response
[43]	Squamous-cell carcinoma in mice	Radiotherapy (γ-rays) and immunotherapy (DC vaccines)	Daylight LED (2.55 mW/cm^2^) for 1 h, 9.18 J/cm^2^)	OR141 (4 and 40 nm/kg)	Subcutaneous SCC7 tumors in C3H or nude mice	Additive effect of DC vaccination peri-radiation
[44]	Triple-negative breast cancer in mice	Chemotherapy (Doxorubicin) and immunotherapy (anti-PD-L1 5 mg/kg)	Not available	AIEgen Dose not available	Cell lines and subcutaneous 4T1 tumors in BALB/c mice	Enhanced tumor suppression
[45]	Intraocular melanoma in mice	Immunotherapy (anti-PD-L1 antibody)	LED (633 nm, 65 mW/cm^2^ for 3 min)	Chlorin (Ce6) Dose not available	Subcutaneous B16F10 tumors in C57B/6 J mice	Enhancement of antitumor immune response

When not specified in the original publication, details on tumor development (subcutaneous or in situ) were not available.

**Table 3:** Summary of studies combining PDT with therapies targeting the TME.

Reference	Target	Cancer Type	PS	Light Source	Sample	Outcome
[55]	TME oxidative stress	Breast in mice	Polymer-encapsulated carbonized hemin nanoparticles (P-CHNPs) (8 mg kg^−1^, 40 μL)	Not available (400–700 nm, 100 mW cm^−2^, 20 min)	Orthotopic breast 4T1 tumor model in BALB/c mice	Better treatment localization, reduced tumor size
[56]	Hypoxia	Cervical in humans	Hemoglobin (Hb)-linked CPNs (8 mg mL^−1^ for 12 h)	Blue light from luminol (375–550 nm)	HeLa cells	Increased oxygen production
[57]	Hypoxia	Cervical in humans	Paclitaxel-loaded human serum albumin nanoparticles conjugated with Azo and Ce6 (RP/CA/PHNPs) (1 mg/mL, 2 days for 4 h)	Laser (670 nm, 150 mW/cm^2^ for 10 min)	HeLa cells	Tumor growth inhibition
[60]	Hypoxia	Colon in mice	Porphyrin-based PS containing methoxy-napthalene (P-nap) 5 mg/kg	Lamp 1200 W (600–720 nm, 20 J cm^−2^, 50 mW cm^−2^)	Subcutaneous CT26 tumors in BALB/c mice	Tumor suppression, increased survival
[61]	Hypoxia	Prostate in mice	Nanoparticles containing PS mTHPC, IR780, and Perfluorooctylbromide (PFOB@IMHNPs) Dose not available	NIR laser (660 nm for 5 min or 808 nm combined with 660 nm for 5 min)	Subcutaneous TRAMP-C1 tumors in C57BL/6 mice	Tumor growth suppression, tumor hypoxia relief
[62]	Hypoxia	Breast in mice	PDA-Pt-CD@RuFc NPs (200 μL, 1 mg mL^−1^, 4 h)	Lasers (450 nm and 808 nm, 1 W cm^−2^)	Mice	Hypoxia reduction, therapeutic effect enhancement
[63]	Hypoxia	Liver in humans	Fe_3_O_4_/Au NCs@LCPAA-TPP nanoplatform (500 μg/mL)	NIR laser (808 nm, 1.0 W·cm^−2^ for 8 min)	Subcutaneous H22 tumors in mice	Hypoxia reduction, induction of apoptosis
[58]	TME vasculature	Colorectal in mice	AFZDA nanoparticles (0.5 mg/kg every other day for 14 days)	NIR laser (808 nm laser at 1.5 W for 3 min)	Subcutaneous CT26 tumors in BALB/c mice	Tumor vessel normalization
[59]	TME vasculature	Breast in mice	Tumor-exocytosed EXO/AIEgen hybrid nanovesicles (DES) (20 mg mL^−1^)	Laser (532 nm, 0.5 W cm^−2^, 5 min)	Subcutaneous 4T1 tumors in BALB/c mice	Hypoxia reduction, tumor growth inhibition
[64]	Mitochondria	Colorectal in humans	MND-IR@RESV (0 mg/mL, 0.3 mg/mL, 0.6 mg/mL, 1 mg/mL)	NIR laser (808 nm at 1 W cm^−2^ for 5 min)	Mice	Induction of tumor cell apoptosis
[65]	ECM components	Colorectal in mice	PCPP (100 μL, 50 mg kg^−1^ for 7 days)	Laser (0.5 W cm^−2^, 10 min)	Subcutaneous CT26 tumors in BALB/c mice	Tumor solid stress and hypoxia reduction

When not specified in the original publication, details on tumor development (subcutaneous or in situ) were not available.
